# The contribution of *citizen science* in the surveillance of wildlife and related arthropods

**DOI:** 10.1017/S0031182023001038

**Published:** 2023-10

**Authors:** Giovanni Sgroi, Nicola D'Alessio, Rachele Vada, Ezio Ferroglio, Joaquin Vicente, Vincenzo Veneziano

**Affiliations:** 1 Department of Animal Health, Experimental Zooprophylactic Institute of Southern Italy, Portici, Italy; 2 Osservatorio Faunistico Venatorio, Naples, Italy; 3 Department of Veterinary Sciences, University of Turin, Italy; 4 Instituto de Investigación en Recursos Cinegéticos, University of Castilla-La Mancha, Ciudad Real, Spain; 5 Department of Veterinary Medicine and Animal Production, University of Naples Federico II, Naples, Italy

**Keywords:** arthropod, census, *citizen science*, parasite, vector-borne pathogens, wildlife

## Abstract

Environmental and anthropogenic factors may significantly affect the diffusion of wild animals, enhancing the interface of human–wildlife interactions and driving the spread of pathogens and vector-borne diseases between animals and humans. However, in the last decade, the involvement of citizens in scientific research (the so-called *citizen science* approach, henceforth abbreviated as CS) provided a network of large-scale and cost-effective surveillance programmes of wildlife populations and their related arthropod species. Therefore, this review aims to illustrate different methods and tools used in CS studies, by arguing the main advantages and considering the limitations of this approach. The CS approach has proven to be an effective method for establishing density and distribution of several wild animal species, in urban, peri-urban and rural environments, as well a source of information regarding vector–host associations between arthropods and wildlife. Extensive efforts are recommended to motivate citizens to be involved in scientific projects to improve both their and our knowledge of the ecology and diseases of wildlife. Following the *One Health* paradigm, collaborative and multidisciplinary models for the surveillance of wildlife and related arthropod species should be further developed by harnessing the potentiality of the CS approach.

## Graphical abstract

**Figure:**
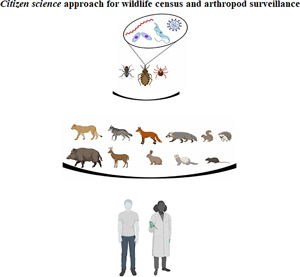

## What does *citizen science* mean? Theoretical considerations

The COVID-19 pandemic has enlightened the world on the risks of spill-over and transmission pathways of zoonotic agents through several interaction phenomena at the human–wildlife interface (One Health High-Level Expert Panel, 2023). This is particularly noteworthy when considering that 70% of zoonoses originate from wildlife (Jones *et al*., 2008; Morse *et al*., 2012) and the increasing density of wild species in urban and peri-urban settlements may act as a trigger for the circulation of zoonotic agents, including those transmitted by arthropods (Wegner *et al*., 2022). Furthermore, several sociodemographic factors, such as agricultural strategies, wildlife management, deforestation and global warming may significantly affect the diffusion of wildlife and arthropod–host associations (Dantas-Torres and Otranto, 2013). These aspects indicate that the monitoring of wildlife populations and their arthropod species is crucial for preventing the potential spread of emerging or re-emerging vector-borne pathogens (VBPs) (Santoro *et al*., 2019; Sgroi *et al*., 2021*a*).

In this scenario, the *citizen science* approach (hereafter CS) facilitates the collection of large-scale data in a cost-effective manner whilst, raising awareness of zoonoses prevention among the general public (Hamer *et al*., 2018). Several interpretations of this approach are to date available in the literature, whereby there are 34 different definitions of CS reported (Haklay *et al*., 2021). For example, UNESCO defines CS as *the participation of a range of non-scientific stakeholders in the scientific process. At its most inclusive and most innovative, CS involves citizen volunteers as partners in the entire scientific process, including determining research themes, questions, methodologies and means of disseminating results* (Haklay *et al*., 2021). Since CS is currently developing so rapidly, the discussions regarding definitions and criteria are so varied, and it is difficult to narrow this down to a single definition (Vohland *et al*., 2021). However, ubiquitous in all definitions is the point that in any CS project, activities are voluntary undertaken by members of the general public who are represented by non-scientific stakeholders interested in a specific problem-solving. Although the practices themselves are much older, CS as a definition originated in the 1990s as a result of the work of scientists such as Alan Irwin and Rick Bonney who recognized the value of data collected by amateur naturalists and its implications in research (Brossard *et al*., 2005). In the last decade, the term CS became extremely popular due to a rise in the number of publications and research projects. As a result, CS is a routine practice supported by social media (acting as ‘brokers’) and public institutions, such as the European Citizen Science Association (ECSA) which promotes the growth of an interdisciplinary and international CS community (Frigerio *et al*., 2018). Therefore, beginning by discussing a symposium during the *Italian Congress of Parasitology 2022* (SoIPa Congress), this review aims to provide an overview on the contribution of CS as a valuable tool in the surveillance of wild animals and their related arthropod species, and highlight its advantages and disadvantages. During the CS approach, mutual exchanges occur between citizens and scientists whereby any information provided by citizens is transferred to scientists who return it in the form of results of public utility. For instance, when citizens collect data (e.g. on the presence of wild animals in an area or the occurrence of arthropods on wildlife), this information is then interpreted and integrated into sources of information for either academia, or public information (e.g. density evaluation of wildlife and risk of pathogen transmission or the presence of vectors in a given environment). Compared to traditional methods, multiple advantages may be considered in studies that involve CS. Firstly, the possibility for scientists to provide public health education and open access to scientific findings for participants. In addition, CS may be a way to spread medical knowledge to geographic areas where such information is poorly available, such as in rural settlements that are commonly characterized by limited public health services (de Vries *et al*., 2018). In these environmental contexts, CS may be a useful tool for supporting hard-to-reach populations (i.e. forestry workers, hunters, farmers), in collaboration with local public institutions (Perry *et al*., 2022; Stufano *et al*., 2022). Secondly, scientists have the opportunity to obtain large amounts of samples and datasets, over wide territories in a cost-effective manner. In fact, the collection from wider geographic areas than possible by small teams of scientists is a common feature of CS studies (Dickinson *et al*., 2010; Hamer *et al*., 2018; Johnson *et al*., 2022; Sgroi *et al*., 2023). However, the involvement of a large number of volunteers does not necessarily increase economic costs by scientific institutions, as most programmes do not pay contributors, as they simply participate because of their enthusiasm and interest in science (Hamer *et al*., 2018; Rafiq *et al*., 2019; Edwards *et al*., 2021; Poh *et al*., 2022). At the same time, the direct collaboration between scientists and citizens promotes transparency and trust between the general public and scientific institutions (Hamer *et al*., 2018). The main advantages of the CS approach outlined above are summarized in Table 1.

**Table 1.** Advantages of *citizen science* approach for citizens and scientists/scientific institutions in the surveillance of wildlife and related arthropods

For citizens	For scientists/scientific institutions
Health education and promotion	Large-scale datasets and amount of samples
Open access to scientific advance	Wide geographic sampling
Trust in public health institutions	Cost-effective research
Citizenship's interest in science	Optimization of research fundings

It is important to note that the challenges and limitations of CS studies may occur in the case that citizens are not properly trained or supported by experts. This can result in misinformation or insufficient volume of data or incorrect procedures in data collection with a consequent wane in public interest and loss of interest in participation over time (Wozniak *et al*., 2015; Laaksonen *et al*., 2017). Other limitations include the potential risk of infection to citizens during collection, transport or transport samples in the field and the need to guarantee anonymity and correct use of their personal information during a CS project. Finally, it is essential to check and verify the robustness and reliability of data collected by volunteers over the life of the programme, especially in long projects (Hamer *et al*., 2018).

## The application of *citizen science* in wildlife census

Contrary to popular belief, census of wildlife goes back centuries through initiatives that today would be described as CS. For example, in late 16th century, the Spanish government created a survey (called ‘relaciones topográficas’), which provided information on the presence of several animal species (Clavero and Revilla, 2014). To date, sightings of wildlife by citizens contribute for obtaining a plethora of information, such as population density (Scott *et al*., 2014), spatial overlap and interactions with humans and companion animals (Goswami *et al*., 2015) and, consequently, help in the prediction of potential routes of pathogen transmission (Lawson *et al*., 2015). Different tools and methods are set up for conducting census activities of wildlife in different countries of the world (Table 2). Most CS projects are based on opportunistic observations of live wild animals through GPS-telemetry (i.e. GPS-transmitters that are fitted to the animals with a collar/ear tag to track their movements) in various environmental contexts, including natural parks or faunal reserve (Rafiq *et al*., 2019; Ostermann-Miyashita *et al*., 2022). Other studies aim to create maps on the distribution of wildlife employing volunteers to fill specific road-killed schedules (ENETWILD-consortium *et al*., 2020; Raymond *et al*., 2021; Heigl *et al*., 2022). A substantial number of CS studies are based on camera trap (or camera capture) surveys, where participants collect images and/or videos of wild species that are further verified by experts (Swanson *et al*., 2016; Caravaggi *et al*., 2018; Lasky *et al*., 2021). Although difficult to apply on a large scale, the camera trap is commonly preferred as an independent, less disruptive and feasible tool for collecting reliable data on wildlife density (ENETWILD-consortium, 2021; ENETWILD-consortium *et al*., 2022*a*).

**Table 2.** *Citizen science* projects based on sightings of wildlife species classified according to taxonomic order

Wildlife species	Contribution of citizen scientists	Country	Reference
Artiodactyla			
Red deer (*Cervus elaphus*)	Sightings reported on website with date and coordinates	Great Britain	Croft *et al*. (2019)
Reindeer (*Rangifer tarandus*) Roe deer (*Capreolus capreolus*)	Sightings reported on website with date and coordinates	Norway	Cretois *et al*. (2021)
Wild boar (*Sus scrofa*)	Sightings reported on questionnaire survey with geographical information	Denmark	Jordt *et al*. (2016)
Carnivora			
African lion (*Panthera leo*)	Assessment of lion tracks reported by cell phone with number of lions, age, sex and predation patterns towards domestic animals	Kenya	Dolrenry *et al*. (2016)
Red fox (*Vulpes vulpes*)	Urban sightings reported on website with date, time and coordinates	Great Britain	Scott *et al*. (2014)
Red fox	Urban sightings reported by phone call, e-mail and online platform, with number of animals, date, time, dens, traces and photographs	Austria	Walter *et al*. (2018)
Wolf (*Canis lupus*)	Sightings based on collection of fecal samples by specific kits with date, coordinates, number of trace marks around the sample and sample freshness value	Finland	Granroth-Wilding *et al*. (2017)
Diprotodontia			
Koala (*Phascolarctos cinereus*)	Sightings reported on website with date, coordinates and geographical information	Australia	Dissanayake *et al*. (2019)
Monotremata			
Short-beaked echidna (*Tachyglossus aculeatus*)	Sightings reported on website with date, time, coordinates and photographs	Australia	Perry *et al*. (2022)
Lagomorpha			
European hare (*Lepus europaeus*) European rabbit (*Oryctolagus cuniculus*)	Sightings reported by camera traps with photographs	Ireland	Caravaggi *et al*. (2018)

The development of Internet and new digital technologies, including smartphone applications and geographical information systems, brought the popularity of CS wildlife sightings in a global dimension. In particular, social media is a key in the success of recruiting of participants to create a network where information is shared between communities of citizens and experts (Chenery *et al*., 2022). These networks are often represented by general social media platforms (e.g. Facebook, Twitter, Flickr) or specific wildlife monitoring channels (e.g. Zooniverse, iNaturalist, EhidnaCSI, iMammalia, MammalNet) (Frigerio *et al*., 2018; ENETWILD-consortium *et al*., 2020, 2022*b*). In particular, Zooniverse is a web platform containing different active projects (e.g. ‘Snapshot Serengeti’ and ‘Chimp & See’) in which volunteers analyse wildlife photographs and videos obtained through camera traps and upload them to an online system (Edwards *et al*., 2021).

While some platforms are generalized, as they allow volunteers to perform multi-species sightings (e.g. iNaturalist) (Callaghan *et al*., 2022), others are focused on tracking and mapping selected species of wildlife, such as ‘Echidna Conservation Science Initiative’ (i.e. EchidnaCSI), a free mobile application to report sightings of European hedgehogs (Perry *et al*., 2022).

Although most platforms can be employed in any country in the world where an Internet connection is available, some are active exclusively in certain geographical areas. This is the case of the ‘Brazilian Wildlife Health Information System’ (i.e. SISS-Geo), a web platform for collaborative monitoring in Brazil, in order to generate preventive measures and computational models for predicting zoonoses of wildlife (Chame *et al*., 2019).

The reliability of CS studies for monitoring wildlife has been ascertained through several investigations. For example, the density of red foxes in urban and peri-urban areas of the UK observed by CS sightings overlapped with the data of a census previously outlined with traditional surveys by scientists (Scott *et al*., 2014). Again, data on the distribution of several wildlife species obtained *via* Flickr users were in accordance with those provided by the National Biodiversity Network (NBN) Atlas in the UK (Edwards *et al*., 2021). In addition, CS projects represent a useful bottom-up approach in low-income and developing countries. In this regard, a community-based conservation study on African lions in Kenya through Maasai warriors (pastoralist men with no formal education) produced reliable data on the demographics and movements of this species, but also direct benefits to participants, such as literacy training, skill enhancement and sensitivity to wildlife (Perry *et al*., 2022). Additionally, CS sightings can be particularly useful in conditions where field activities by experts are not possible or allowed. For example, long-term restrictions during the COVID-19 lockdown promoted increased movements of wild animals near urban settlements (Manenti *et al*., 2020), together with the inability to run traditional surveillance plans of wildlife; however, during this period, CS sightings continued, confirming their value and utility (Stenhouse *et al*., 2022). Lastly, CS projects may be a cost-effective strategy for wild populations surveillance, as demonstrated by a study where photographs by visitors proved to be a cheaper investigation framework compared to camera trap, call-in station and spoor survey for monitoring the circulation of animals in protected areas (i.e. natural parks and faunal reserves) (Rafiq *et al*., 2019).

## *Citizen science* and arthropod surveillance in wildlife

The CS approach in arthropod surveillance frequently relies on the involvement of specific stakeholder groups (e.g. hunters), including in sample collection activities. For example, CS projects where citizens collect hard ticks (Ixodidae) from wildlife are useful to determine which animal species harbour a given tick species (Table 3) and, consequently, to predict which VBPs potentially circulate in a certain area. Data on vectors and VBPs in wildlife are made available *via* CS, such as arthropod–host associations (Heylen *et al*., 2017), reservoirs of zoonotic infections (Raizman *et al*., 2013), target organ of infection in the host (Zinck and Lloyd, 2022) and risk of infection for animals and humans in a geographic area (Nieto *et al*., 2018; Sgroi *et al*., 2021*b*, 2022; Stufano *et al*., 2022).

**Table 3.** *Citizen science* surveys investigating the association between arthropods and wildlife according to taxonomic order

Wildlife species	Arthropod species	Contribution of citizen scientists	Country	Reference
Artiodactyla				
White tailed deer (*Ococoileus virginianus*)	*Ixodes scapularis*, *Amblyomma americanum*	Assistance in carcass inspection for tick removal at hunter check stations	USA	Apperson *et al*. (1990)
White tailed deer	*Ixodes scapularis*, *Lipoptena cervi*	Ectoparasite removal and collection of animal data (county, township, date and time of harvest) at hunter check stations	USA	Poh *et al*. (2020)
White tailed deer	*Lipoptena cervi*	Ked removal by hunters with a specific ked-kit	USA	Evans *et al*. (2021)
Fallow deer (*Dama dama*) Red deer (*Cervus elaphus*) Roe deer (*Capreolus capreolus*)	*Lipoptena fortisetosa*	Ked removal and collection of animal skin samples by trained hunters	Italy	Andreani *et al*. (2021)
Red brocket deer (*Mazama americana*)	*Amblyomma dissimile*	Tick removal by hunters	Trinidad	Sameroff *et al*. (2021)
Wild boar (*Sus scrofa*)	*Dermacentor marginatus*, *Dermacentor reticulatus*, *Ixodes ricinus*, *Haemaphysalis concinna*	Tick removal by licensed hunters	Hungary	Hornok *et al*. (2022)
Carnivora				
Coyote (*Canis latrans*)	*Dermacentor variabilis*	Tick removal by hunters and volunteers with collection kit and collection of animal data (age, sex, date, coordinates and tick attachment site)	USA	Hertz *et al*. (2017)
Otter (*Lutra lutra*)	*Ixodes hexagonus*	Tick removal by volunteers, amateur entomologists, wildlife charities and collection of animal data (date, coordinates and tick attachment site)	USA	Cull *et al*. (2018)
Eulipotyphla				
Hedgehog (*Erinaceus europaeus*)	*Ixodes hexagonus*	Tick removal by volunteers and wildlife charities and collection of animal data (date and coordinates)	Great Britain	Hansford *et al*. (2022)

In addition to ticks, citizens can participate in the collection of dead animals that are then tested by scientists to assess their positivity for VBPs (Zinck and Lloyd, 2022). This method has been applied in a study in which rodent carcasses were collected by the public and delivered to scientists for testing different VBPs, proving that the jumping mouse and eastern deer mouse were the main reservoirs of Borrelia burgdorferi sensu lato complex in Canada (Zinck and Lloyd, 2022). This study also investigated the different target organs of infection by *B. burgdorferi* s. l. compared to the emerging *Borrelia miyamotoi*. In particular, the study found that *B. burgdorferi* s. l. was able to spread in the hosts to a single tissue, whereas *B. miyamotoi* in multiple organs. In addition, this survey has suggested a potential vertical transmission (i.e. transplacental) of *B. miyamotoi* in mammals, being likely able to rapidly spread within wildlife populations (Zinck and Lloyd, 2022).

Furthermore, the association between keds (Hippoboscidae) and wildlife (Table 3) is relevant due to the suspected role of these insects in the transmission of VBPs. For example, *Lipoptena cervi* parasitizes both humans and ungulates (i.e. fallow deer, red deer, roe deer, white-tailed deer) and has been indicated as a potential candidate to vector bacterial and parasitic agents, although experimental studies are needed to clarify its role (Bezerra-Santos and Otranto, 2020).

Most CS projects are state-registered, thus scientists directly support citizens in field activities. This approach is used by a national tick surveillance plan in the UK by the Health Security Agency, who supports volunteers in tick sampling and assessing seasonal activity and host association of arthropods (Cull *et al*., 2018; Hansford *et al*., 2022).

In other CS studies, ready-to-use collection kits (i.e. combs, tweezers, vials with 70% ethanol) are given by scientists to groups of hunters who attend training courses and follow instructions to collect, store and deliver ectoparasites obtained from their harvests (Hertz *et al*., 2017), often using check stations, public hunting lodges or private slaughterhouses (Poh *et al*., 2022). An example of large-scale sampling of ectoparasites directly run by citizens is the Project Acari, where participants use a custom-designed ‘tick kit’ that includes a tick card, clear stickers to attach ticks on the card and a biohazard bag with plastic envelope for return shipping (Chauhan *et al*., 2020). Up to date, this study has involved 900 citizens and obtained 322 tick cards, with a total of 2417 ticks, from 32 states of the USA. The use of tick-cards could be used in the surveillance of ectoparasites of wild animals, by the involvement of hunters or other non-scientific stakeholders, such as wildlife conservationists and naturalists. Similarly, Evans *et al*. (2021) has demonstrated the efficacy of a ‘ked-kit’ delivered to hunters for the collection of keds (*L. cervi*) from the white-tailed deer in the USA. Details on CS studies characterized by a direct engagement of citizens in the collection of ectoparasites from wild animals and related information are reported in Table 3.

One of the crucial points of CS projects focused on arthropods is the scientific quality and reliability of data provided by citizens who have little experience in this field. Although citizens are traditionally not educated about arthropods and VBPs initially, a coherent and well-designed project can significantly improve their theoretical and practical knowledge. For example, it has been demonstrated that as soon as deer hunters become familiar with ectoparasites, they are able to collect samples from a greater variety of geographic areas than researchers (Evans *et al*., 2021), probably due to their deeper knowledge of local rural contexts compared to scientists. Again, in a CS study, the prevalence of *Trypanosoma cruzi* in kissing bugs (Hemiptera) from wildlife nests of Texas (i.e. 63%) was similar to that retrieved in the same geographical areas by traditional research studies (i.e. 50.7%) (Curtis-Robles *et al*., 2015). This suggests that the scientific reliability of a project does not depend on whether it is a CS or traditional research but rather on the solid planning of the study itself.

Despite its potential, CS has limitations. For example, species such as kissing bugs, biting flies and mosquitoes do not remain after feeding on wildlife carcasses which makes the collection of these arthropods difficult (Poh *et al*., 2022). This gap may be partially bridged by identifying the transmitted pathogens rather than the arthropod itself in a given animal species. This approach has been employed to assess the presence of Triatominae (e.g. *Triatoma* spp., *Panstrongylus* and *Rhodnius* spp.) on the white-tailed deer in Texas, to detect *T. cruzi* DNA in the animal's heart (Gunter *et al*., 2018). However, while detecting a VBP provides information on the prevalence of infection in wildlife populations, it does not represent a vector surveillance, neither evidence for host–vector interactions. Finally, the morphological identification of arthropods at species level requires skilled experience; therefore, in all CS projects, the final identification of ectoparasites is run by experts (Johnson *et al*., 2022).

## Future considerations

As a result of the climate challenges, human-induced environmental changes and restriction of natural habitats, the composition and urbanization of wildlife communities may inadvertently enhance human–wildlife interactions and drive the rise of emerging infectious diseases in humans, companion and farm animals (Sgroi *et al*., 2021*b*).

In this scenario, CS could be more beneficial than ever, thus this review has outlined examples on how this approach represents a impactful model in the surveillance of wildlife and their related arthropods, with respect to its disadvantages. The main limitation of CS is motivating citizens to participate. However, once this issue has been addressed, and the technique of engaging a large number of citizens in science has been improved upon, then the outcome will also be greater.

Following the *One Health* paradigm, collaborative and multidisciplinary workflows in the surveillance of wildlife and related arthropods should be further developed by harnessing the power of citizens (Lawson *et al*., 2015). For this reason, the top challenge for research institutions and intergovernmental agencies is to open public accession lines to impactful and long-term CS projects. A deeper and coordinated effort is needed by scientists, stakeholders, local veterinary services and rescue centres, to improve the knowledge and training of citizens on ecology and wildlife disease, but also for extending the surveillance to non-communicable pathogens and neglected hosts (ENETWILD-consortium *et al*., 2023).

To conclude, the involvement of the media and politicians in motivating and raising awareness of the importance of wildlife and disease management would surely hugely benefit the development of strategic wildlife monitoring plans at an international level.

## Data Availability

Data availability is not applicable to this article as no new data were created or analysed in this study.
